# SV-MeCa: an XGBoost-based meta-caller approach for structural variant calling from short-read data

**DOI:** 10.1186/s12859-025-06246-6

**Published:** 2025-08-20

**Authors:** Rudel Christian Nkouamedjo Fankep, Arda Söylev, Anna-Lena Kobiela, Jochen Blom, Corinna Ernst, Susanne Motameny

**Affiliations:** 1 Center for Familial Breast and Ovarian Cancer, Center for Integrated Oncology (CIO), University of Cologne, Faculty of Medicine and University Hospital Cologne, Cologne, Germany; 2 Institute for Medical Biometry and Bioinformatics, Heinrich Heine University Düsseldorf, Medical Faculty, Düsseldorf, Germany; 3 Center for Digital Medicine, Heinrich Heine University Düsseldorf, Düsseldorf, Germany; 4 Bioinformatics and Systems Biology, Justus Liebig University Gießen, Gießen, Germany; 5 Cologne Center for Genomics, University of Cologne, Medical Faculty, Cologne, Germany; 6 West German Genome Center - Cologne, University of Cologne, Cologne, Germany

**Keywords:** Structural variants, Variant calling, Whole-genome sequencing, Meta-caller

## Abstract

**Background:**

Calling structural variants (SVs), i.e., genomic alterations of $\ge$50bp, from whole genome short-read data remains challenging, as existing callers are known to lack accuracy and robustness. Therefore, meta-caller approaches combining the results of multiple standalone tools in a consensus set of reported SV calls, are widely used. Here, SV-MeCa (Structural Variant Meta-Caller) is presented, the first SV meta-caller incorporating variant-specific quality metrics from individual VCF outputs, rather than relying solely on number and combination of tools supporting consensus SV calls. In addition, SV-MeCa offers a suitable score to rank obtained consensus SV calls according to evidence of representing true positive calls, i.e., real-world variants.

**Results:**

SV-MeCa applies seven standalone SV callers and merges resulting deletion and insertion calls into a union VCF file using SURVIVOR. For each entry in the SURVIVOR-generated consensus, caller-specific quality measures are extracted from corresponding standalone VCF files, and serve as input for an either deletion- or insertion-specific XGBoost decision tree classifier, which was previously trained on the HG002 SV benchmark data provided by the Genome in a Bottle consortium. The SV-MeCa XGBoost models assign a probability to (consensus) SV calls to represent true positive calls, which can be used for ranking the final output according to evidence. Performance of SV-MeCa and four previously published meta-caller approaches were evaluated based on autosomal SV calls in samples curated by the Human Genome Structural Variation Consortium, Phase 2. With regard to F$_1$ scores, which were 0.58 on average for deletions and 0.42 on average for insertions, SV-MeCa outperformed the other meta-callers. With regard to precision, only ConsensuSV achieved higher values (0.97 versus 0.64 on average for deletions, 0.75 versus 0.53 on average for insertions), and with regard to recall, SV-MeCa was outperformed exclusively by Meta-SV for deletions (0.55 versus 0.53).

**Conclusions:**

SV-MeCa, publicly available at https://github.com/ccfboc-bioinformatics/SV-MeCa, outperforms existing SV meta-caller approaches by taking variant-specific quality measures into account. Moreover, due to the XGBoost prediction probabilities serving as scores, the output of SV-MeCa can be continuously adjusted to user needs in terms of sensitivity and precision.

**Supplementary Information:**

The online version contains supplementary material available at 10.1186/s12859-025-06246-6.

## Background

Structural variants (SVs) are usually defined as genomic alterations of the types deletions, duplications, insertions, inversions, or translocations affecting $\ge$50bp [1]. Detection of SVs based on short-read data requires more sophisticated algorithms compared to single nucleotide variants and short indels discovery [2, 3]. Current approaches typically pursue one or a combination of four main strategies. Read depth- or read count-based approaches are commonly used for the identification of copy number variants (CNVs), i.e., large deletions or duplications having significant effects on observed read depth in the affected genomic region. On the other hand, read-pair methods consider insert-size and orientation of paired-end reads, whereas the split read-based approaches identify the start and stop positions of putative SVs by scanning for partially mapped (i.e., split) reads. Furthermore, SVs can also be detected based on *de novo* assembly.

Apart from the inherent limitations of short-read sequencing, the accuracy and robustness of current SV callers limit our ability to discover SVs effectively. Thus, we are not able to detect the full range of existing SVs [2–5]. Meta-caller approaches, combining the results of multiple standalone tools have been developed and shown to achieve improved performance [2, 6] in this regard. However, all the currently available approaches applicable to short-read whole-genome sequencing (WGS) data have their specific drawbacks and limitations. MetaSV expects already generated VCF files as input, leaving the execution of standalone SV callers to the responsibility of the user [7]. Furthermore, it is unable to extract insertion calls properly from VCF input (except for Pindel [8]) and therefore includes its own split read- and assembly-based method for insertion detection, which in turn makes the intended meta-approach obsolete. In contrast, Parliament2 [9] and ConsensuSV [10] provide ready-to-use SV meta-caller pipelines that start from raw FASTQ files and allow an SV meta-calling with minimal user effort. Both tools, however, predominantly assess the evidence of an SV call being true positive (TP) based on the consistency with which it is detected by multiple standalone callers or their combination. Parliament2 reports quality values of consensus SV calls based on pre-computed precisions per combination of involved standalone SV callers, SV type and SV length range [9]. ConsensuSV considers and reports only overlapping SV calls of the same type originating from at least a pre-defined number of standalone callers [10]. Similarly, FusorSV uses pre-computed cut-offs per SV caller combination and SV length range to determine the final output of consensus SV calls [11]. Also, the recently published VISTA tool creates the final output from the union of all SV calls of a pre-defined set of standalone callers per SV type and length range [12]. All these strategies share the common fundamental limitation that individual SV calls enter the process of building a final consensus list of putative SVs as virtually binary encodings of their presence. SV calls from only one or very few standalone tools are generally considered to have a low probability to represent TP events, regardless of the qualities assigned to them. Inversely, consistent calls from many approaches are always associated with a high probability of representing TPs, even if consistently reported as low confidence calls.

Here we present SV-MeCa (Structural Variant Meta-Caller), which overcomes these limitations of consensus SV call quality assessment by incorporating variant-specific quality metrics from individual SV caller’s VCF outputs. In addition, SV-MeCa provides a suitable and continuous score to rank consensus SV calls according to evidence of representing TP events.

## Methods

### General approach of SV-MeCa

**Fig. 1:**
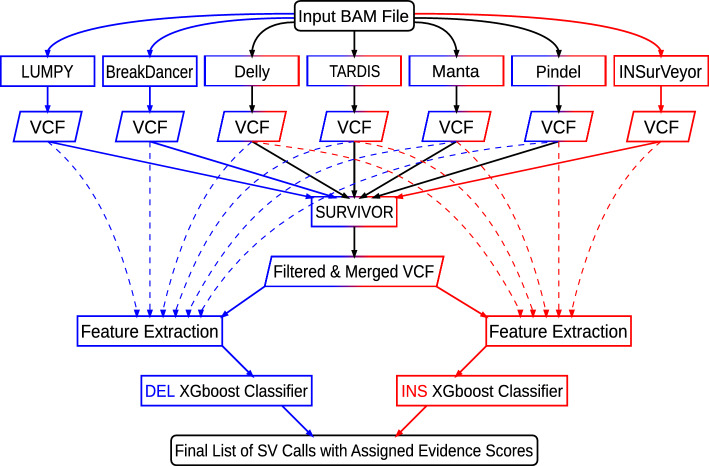
Summary flowchart of the SV-MeCa approach. VCF output files from seven standalone structural variant (SV) callers (BreakDancer, LUMPY, Delly, TARDIS, Manta, and Pindel for deletions; Delly, TARDIS, Manta, Pindel, and INSurVeyor for insertions including duplications) are processed. Steps specific to deletions are shown in blue, whereas steps specific to insertions including duplications are shown in red

Starting from a paired-end short-read WGS BAM file including split read information, SV-MeCa incorporates seven SV callers, namely BreakDancer [13], Delly [14], LUMPY [15], Manta [16], Pindel [8], TARDIS [17, 18], and INSurVeyor [19] (Fig. 1). Resulting VCF files are filtered for insertions (including duplications) and deletions with length $\ge$50bp. Subsequently, deletions reported by LUMPY, Manta, Delly, Pindel, and TARDIS, and insertions reported by LUMPY, Manta, Delly, TARDIS, and INSurVeyor are merged into a single VCF file using SURVIVOR [20]. For each entry in the SURVIVOR-generated consensus, caller-specific quality measures are extracted from the corresponding VCF files and serve as input for an either deletion- or insertion-specific XGBoost (Extreme Gradient Boost) decision tree classifier [21]. These pre-trained XGBoost models assign probabilities to (consensus) SV calls to represent TPs, which are then used for the ranking of the final output.

### Benchmarks

We employed the HG002 (NA24385) benchmark data provided by the Genome in a Bottle (GIAB) consortium [22] and SVs identified in the children of three parent–child trios (HG00514, HG00733, NA19240) by the Human Genome Structural Variation Consortium, Phase 2 (HGSVC2) [23, 24], Freeze 3, as ground truths for model development and evaluation. Details on data preparation are given in Supplementary Methods.

For the creation of the training data, the GIAB HG002 Tier1 v0.6 SV benchmark [22], including curated SVs in challenging medically relevant genes [25], was restricted to autosomal variants with length $\ge$50bp in heterozygous or homozygous genotype. We aligned 250bp Illumina paired reads of HG002 against the GRCh37 reference genome, resulting in a mean coverage of 52x. In order to make it more consistent with scientific and clinical practice, we downsampled the mean coverage to 35x.

For evaluation, we employed data mapped to the GRCh38 reference genome exclusively. To assess performance of standalone SV callers and SV-MeCa for SVs in challenging medically relevant genes [25], we used 150bp paired-end PCR-free WGS data, which was already downsampled to approximately 30x and provided by the Human Pangenome Reference Consortium^1^ (HG002-CMRG).

In addition, we retrieved paired-end PCR-free WGS data from HG00514, HG00733, and NA19240 from the 1000 Genomes International Genome Sample Resource (IGSR) data portal^2^ to compare the performance of SV-MeCa against standalone SV callers and existing meta-caller approaches on autosomal chromosomes. Mean coverages of HG00514, HG00733, and NA1940 ranged between 30x and 31x, and read length was 150bp.

### Standalone structural variant callers

Various tools for SV calling based on short-read WGS data in SAM/BAM format were eligible for inclusion in the SV-MeCa workflow. We chose the established tools BreakDancer [13], Delly [14], LUMPY [15], Manta [16], Pindel [8], and TARDIS [17, 18], as well as INSurveyor, which was specifically developed for calling insertions [19]. We merged individual SV calls of identical type into consensus calls using the merge utility of SURVIVOR v1.0.7 [20]. A short description of each tool, settings for the SV calling procedure and running SURVIVOR, are given in Supplementary Methods.

Finally, for XGBoost classifier training, we compared SURVIVOR’s output against the GIAB HG002 benchmark VCF using the Truvari v3.5.0 bench utility [26] with default parameters, but restricted to autosomes, specifically autosomal regions specified in GIAB Tier v0.6 BED, and setting –pctseq to 0 to avoid sequence comparisons.

### Model features

For each SV type, we only considered features originating from indidividual VCF output files, if the corresponding SV caller achieved values of $F_1 > 0.1$ based on the 35x GIAB HG002 reference (Table 1), with

1 $$\begin{aligned} F_1 = \frac{2\:\# \text {TP}}{2\:\# \text {TP} + \#\text {FP} + \# \text {FN}} \end{aligned}$$

and #TP denoting the number of TP, #FP the number of false positive, and #FN of false negative predictions. Therefore, we used quality metrics retrieved from the VCF output of BreakDancer, Delly, LUMPY, Manta, Pindel, and TARDIS only for the deletion-specific model, and of Delly, Manta, Pindel, TARDIS, and INSurVeyor only for the insertion-specific model.

We provide details on extraction and transformation of features that are retrieved from SURVIVOR and individual VCF output files in Supplementary Methods, and a summary of all covariates considered in model development in Supplementary Table S1.

The selection of features to be incorporated into the XGBoost classifiers aimed to provide a model applicable to input data within a broad range in terms of read lenghts and observed sequencing coverages.

To achieve this, we normalized the quality metrics that refer to (local) coverage or counts of supporting reads by observed mean coverage (Supplementary Table S1). Further, we also normalized the average edit distances of reads supporting insertion calls (so-called stable reads), as reported by INSurVeyor, by read length.

To assess the dependence of reported quality scores on mean coverage, we analyzed corresponding values from concordant calls based on the 52x and the 35x 250bp HG002 sample (see Supplementary Methods, Supplementary Figures S1 and S2 for details).

Finally, we applied a correlation-based feature selection to determine the initial set of covariates to be considered for the SV-MeCa XGBoost classifier training (Supplementary Methods).

This filtering strategy resulted in a set of 35 covariates as initial input for XGBoost training of the deletion-specific SV-MeCa model, namely 3 derived from BreakDancer’s VCF output, 10 from Delly, 7 from LUMPY, 7 from Manta, 3 from PINDEL, and 4 from TARDIS. To train the insertion-specific SV-MeCa model, 36 features were initially included, 6 of which were specific to Delly, 18 to INSurVeyor, 6 to Manta, 3 to Pindel, and 2 to TARDIS (Supplementary Table S1).

### XGBoost classifier training

For each SV type, two classifier models were trained on autosomal 35x GIAB HG002 data and evaluated, namely the final SV-MeCa XGBoost model, hereafter referred to as full model (FM), including the final subset of covariates to consider, and a basic model (BM) incorporating SV length and binary encoding of the presence of corresponding SV calls per standalone caller exclusively, to demonstrate the benefit of incorporating VCF-derived, SV call-specific quality metrics.

Python implementation of XGBoost Classifier,^3^ an ensemble learner of decision trees, was chosen as supervised learning approach and subsequent predictor due to its scalability, robustness and handling of sparse data [21]. The algorithm works by iteratively fitting decision trees on the residuals of the previous tree, and adjusting the weights of the misclassified samples to prioritize their correct classification in subsequent trees. For the final prediction, the results of individual trees in the entire ensemble are combined.

We employed the exact greedy method with default gbtree booster for individual tree construction, and according to the aim of generating a binary classifier, we used binary logistic regression as objective. Details on hyperparameter tuning are given in Supplementary Methods.

We evaluated model performances based on precision $\frac{\#\text {TP}}{\#\text {TP}+\#\text {FP}}$, recall $\frac{\#\text {TP}}{\#\text {TP}+\#\text {FN}}$ and $F_1$ (Eq. 1). SV calls were predicted as positive, if the classifier’s prediction probability was $\ge$0.5. For calculating #FN, also those SVs were included that were not called by any of the standalone callers applied.

If covariates in trained FMs showed zero feature importance (due to the feature_importances_ attribute), which is defined as the relative contribution of the corresponding feature to the model (gain) per default, we repeated hyperparameter tuning and model training with the corresponding covariates discarded.

### SV-MeCa Implementation

SV-MeCa is implemented as a Nextflow v23.10 [27] pipeline, and uses a conda v23.1.0 environment to ensure consistent package management. It is available as a Docker container, ensuring streamlined deployment, portability, and reproducibility.

SV-MeCa expects BAM or VCF files as input. Given BAM files, SV-MeCa employs the CollectWGSMetrics utility of GATK v4.3 to assess read coverages. Read lengths are retrieved from BreakDancer’s CFG output. If VCF files are given as input, mean coverage and read length are required as additional arguments.

SV-MeCa provides one VCF per executed SV caller and a final consensus VCF as output. In the consensus VCF, prediction probabilities, i.e., final ranking scores, are encoded as QUAL values. Furthermore, to enable straightforward lookup of originating calls in standalone VCFs, all identifiers contributing to a joined call are listed in the final consensus VCF.

### Evaluation

We evaluated the performance of SV-MeCa and four other meta-caller approaches for SV calling, namely ConsensuSV [10], MetaSV [7], Parliament2 [9], and recently published VISTA [12], using the samples HG00514, HG00733, and NA19240 from HGSVC2, considering precision, recall and $F_1$ score. Details on execution of the meta-caller approaches can be found in Supplementary Methods. We were unable to resolve the dependencies of the requirements for running FusorSV [11]. Thus, FusorSV could not be included in our evaluation. Further, the VISTA framework provides only pre-trained models and corresponding scripts for deletions, and hence, was only included in our evaluation for deletions.

## Results

### Standalone structural variant caller results

From the GIAB HG002 reference, 9264 SVs (4051 deletions and 5213 insertions) passed all preprocessing filters and were further considered in SV-MeCa model development and evaluation. Considering the 35x HG002 sample, 20.83% (844/4051) of the deletions and 51.69% (2695/5213) of the insertions were not included in the SURVIVOR-generated union of the results of all standalone SV callers under investigation (Table 1). All SV callers performed noticeably better for deletions than for insertions, with $F_1$ values for deletions ranging from 0.33 (BreakDancer) to 0.78 (Manta) and for insertions from <0.01 (BreakDancer) to 0.31 (Pindel), respectively 0.50 for the insertion-only caller INSurVeyor.

Considering the 52x HG002 sample, 18.04% (731/4051) of the deletions and 44.91% (2341/5213) of the insertions were not included in the SURVIVOR-generated union of the results of all standalone SV callers under investigation (Supplementary Table S2). However, besides #TP, also the amounts of false positive calls in the SURVIVOR union increased with the increase in sequencing coverage from 35x to 52x, namely from 23.16% (967/4174) to 50.11% (3335/6655) for deletions and from 33.24% (1254/3772) to 56.64% (3751/6623) for insertions (Table 1 and Supplementary Table S2), resulting in decreased $F_1$ values (for deletions: 0.78 versus 0.62, for insertions: 0.56 versus 0.49).

**Table 1:** Performance of standalone structural variant callers on 35x HG002

		#TP	#FP	#FN	Precision	Recall	$F_1$
BreakDancer	DEL	902	581	3149	0.61	0.22	0.33
	INS	2	7	5211	0.22	$<0.01$	$<0.01$
Delly	DEL	2251	402	1800	0.85	0.56	0.67
	INS	352	45	4861	0.89	0.07	0.13
INSurVeyor	INS	1800	131	3413	0.93	0.35	0.50
LUMPY	DEL	2055	132	1996	0.94	0.51	0.66
	INS	22	15	5191	0.59	$<0.01$	$<0.01$
Manta	DEL	2649	97	1402	0.96	0.65	0.78
	INS	571	55	4642	0.91	0.11	0.20
Pindel	DEL	2617	504	1434	0.84	0.65	0.73
	INS	1061	668	4152	0.61	0.20	0.31
TARDIS	DEL	1278	28	2773	0.98	0.32	0.48
	INS	414	471	4799	0.47	0.08	0.14
Union SV calls	DEL	3207	967	844	0.77	0.79	0.78
	INS	2518	1254	2695	0.67	0.48	0.56

Summary of numbers of true positive (#TP), false positive (#FP), and false negative (#FN) structural variant (SV) calls, and resulting precision, recall and $F_1$ value (Eq. 1), given the Genome in a Bottle (GIAB) 35x HG002 reference data set per standalone caller and for the (from SURVIVOR consensus derived) union of all calls, subdivided by deletions (DEL) and insertions including duplications (INS). Note that INSurVeyor can only call insertions, and for the evaluation of Delly and INSurVeyor as standalone approaches, only high confidence calls were considered (see Supplementary Methods)

### Model parameters

We show hyperparameters retrieved by grid search in Supplementary Table S3. For the deletion-specific FM, a feature importance of zero was reported for 4 of the 35 covariates initially included in XGBoost training, as well as for 2 of the 36 covariates initially included in the insertion-specific FM training (Supplementary Table S1). Omitting these features did not affect the results of hyperparameter tuning (Supplementary Table S3), nor training accuracies (Supplementary Table S4).

Tables 2 and 3 present comprehensive summaries of the features considered for each standalone SV caller in the final SV-MeCa approach per SV type.

**Table 2:** Densed summary of quality metrics considered by the deletion-specific SV-MeCa XGBoost classifier

	BreakDancer	Delly	LUMPY	Manta	Pindel	TARDIS
No. of supporting read pairs	$\bullet$			$\bullet$		$\bullet$
Microhomology length		$\bullet$		$\bullet$	$\bullet$	
Quality assigned (QUAL, CNVL)		$\bullet$		$\bullet$		$\bullet$
Strand bias	$\bullet$		$\bullet$			
Genotype quality		$\bullet$	$\bullet$			
Local read depth		$\bullet$			$\bullet$	
No. of supporting SRs		$\bullet$				$\bullet$
Variant fraction / Genotype			$\bullet$		$\bullet$	
SR consensus alignment quality		$\bullet$				
Consensus sequence entropy		$\bullet$				
PRECISE vs IMPRECISE			$\bullet$			

Considered metrics per standalone structural variant caller for deletions. A detailed listing of the overall 31 features of the deletion-specific model is given in Supplementary Table S1. SR: Split read

**Table 3:** Densed summary of quality metrics considered by the insertion-specific SV-MeCa XGBoost classifier

	Delly	INSurVeyor	Manta	Pindel	TARDIS
Microhomology length		$\bullet$	$\bullet$	$\bullet$	
No. of supporting SRs		$\bullet$	$\bullet$		$\bullet$
Quality assigned (QUAL, CNVL)	$\bullet$				$\bullet$
No. of supporting read pairs		$\bullet$	$\bullet$		
PRECISE vs IMPRECISE		$\bullet$	$\bullet$		
Local read depth		$\bullet$		$\bullet$	
SR consensus alignment quality	$\bullet$				
Consensus sequence entropy	$\bullet$				
Genotype quality			$\bullet$		
Variant fraction / Genotype					$\bullet$

Considered metrics per standalone structural variant caller for insertions. A detailed listing of the overall 34 features of the insertion-specific model is given in Supplementary Table S1. Reported strand biases were not considered by the model. SR: Split read

Within the 31 covariates of the deletion-specific FM, the (normalized) quality values QUAL from Manta showed by far the highest feature importance, i.e., relative contribution to the model, directly followed by (normalized) counts of paired reads provided by Delly, variant fraction estimates from Pindel, and allele balance reported by LUMPY (Supplementary Figure S2). The covariate with the highest contribution provided by TARDIS, namely reported quality CNVL, was ranked seventh. The SV length and all features retrieved from BreakDancer were listed within the eight features with lowest feature importances.

Of the 34 covariates of the insertion-specific FM, INSurVeyor provided the two features with highest feature importances, directly followed by Manta’s (normalized) count of supporting paired reads, TARDIS’ (normalized) count of supporting split reads, and Pindel’s local coverage estimate (Supplementary Figure S3). The feature importances of the four covariates provided by Delly were consistently below the one of SV length, which was ranked 12th.

### Model evaluation

**Table 4:** Performance of SURVIVOR union, according to curated, GRCh38-based structural variant references

		Reference	SURVIVOR union			
			#TP	#FP	#FN	$F_1$
HG002-CMRG	DEL	98	45	68	53	0.43
	INS	118	29	46	89	0.30
HG00514	DEL	8583	4938	11460	3645	0.40
	INS	13441	5509	18565	7932	0.29
HG00733	DEL	8771	5037	11958	3734	0.39
	INS	13603	5633	22277	7970	0.27
NA19240	DEL	10274	5832	12434	4442	0.41
	INS	15451	6448	21430	9003	0.30

Summary of counts of true positive (#TP), false positive (#FP), and false negative (#FN) structural variant calls, as well as resulting $F_1$ values, according to GRCh38-based structural variant reference in challenging medically relevant genes of sample HG002 (HG002-CMRG) and HGSVC2 reference in 1000 Genomes samples HG00514, HG00733 and NA19240. Values of #TP, #FP and #FN for deletions (DEL) refer to the SURVIVOR-derived union of SV calls retrieved from BreakDancer, Delly, LUMPY, Manta, Pindel, and TARDIS. Corresponding values for insertions (INS) refer to the SURVIVOR-derived union of SV calls retrieved from Delly, INSurVeyor, Manta, Pindel, and TARDIS

The number of reference deletions and insertions (including duplications) per HGSVC2 benchmark sample (Table 4) was more than double compared to the HG002 GIAB reference (Table 1).

Values of $F_1$ achieved by the SURVIVOR union (Table 4) of deletion calls retrieved from BreakDancer, Delly, LUMPY, Manta, Pindel, and TARDIS ranged from 0.39 to 0.43. Values of $F_1$ achieved by the SURVIVOR union of insertion (including duplication) calls retrieved from Delly, INSurVeyor, Manta, Pindel, and TARDIS ranged from 0.27 to 0.30. Numbers of exclusive calls, i.e., TP calls that were uniquely identified by a single tool, are shown for HGSVC2 reference samples in Supplementary Figure S5.

**Fig. 2:**
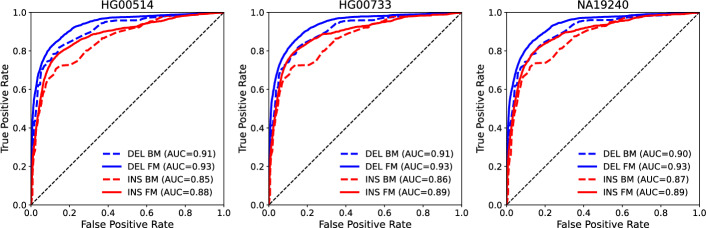
Receiver operating characteristic (ROC) curves of the basic and full (i.e. SV-MeCa) models (BM/FM). Curves for deletions (DEL) are shown in blue, curves for insertions including duplications (INS) are shown in red

The BMs and the final FMs included in SV-MeCa were applied to the deletion and insertion calls in the SURVIVOR union per HGSVC2 sample in order to evaluate XGBoost model performances. The BMs achieved model accuracies between 0.71 and 0.72 (AUC = 0.90 – 0.91) for deletions and between 0.63 and 0.65 (AUC = 0.85 – 0.87) for insertions (Supplementary Table S5). In contrast, the FMs incorporated into SV-MeCa achieved accuracies ranging from 0.79 to 0.81 (AUC = 0.93 each) for deletions and from 0.76 to 0.80 (AUC = 0.88 – 0.89) for insertions. Corresponding ROC curves are shown in Fig. 2.

**Fig. 3:**
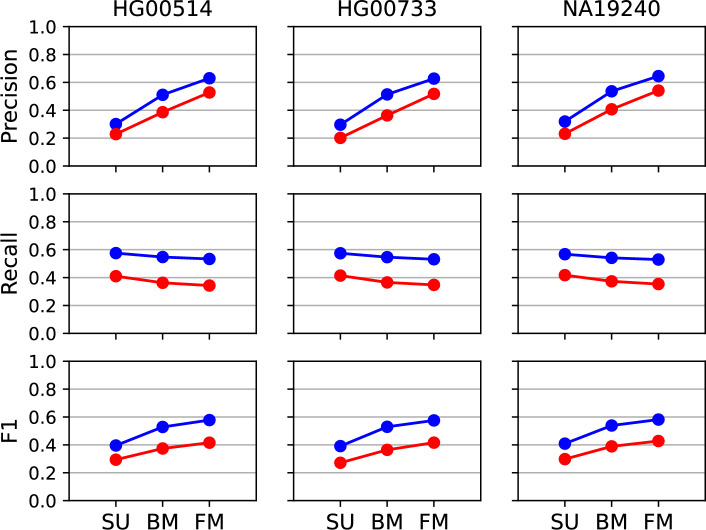
Performance comparison for SURVIVOR union, basic and full (SV-MeCa) model. Precision, recall and F1 value (Eq. 1) for reference samples HG00514, HG00733 and NA19240 under consideration of the SURVIVOR union (SU), the basic model (BM) and the final full model (FM) included in the SV-MeCa approach for deletions (shown in blue) and insertions including duplications (shown in red)

Overall performance metrics of the BMs and the FMs under inclusion of the entire set of false negative calls, i.e., reference SVs that were not detected by any of the incorporated standalone callers, are visualized in Fig. 3 and listed in Supplementary Table S6. SV-MeCa’s FMs showed slightly decreased recall, but outperformed the BMs with regard to precision and $F_1$.

### Comparison to standalone structural variant callers

**Fig. 4:**
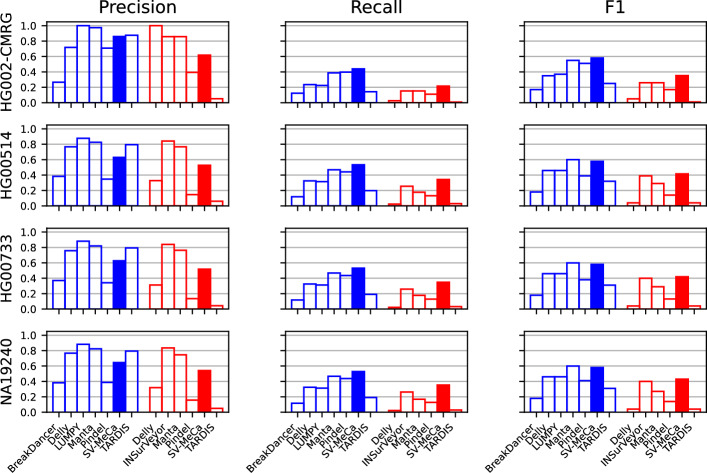
Performance comparison for standalone callers and SV-MeCa. Precision, recall and F1 value (Eq. 1) for GRCh38-based references, namely structural variants in challenging medically relevant genes (HG002-CMRG) and HGSVC2 samples HG00514, HG00733 and NA19240, stratified by deletions (shown in blue) and insertions including duplications (shown in red)

We compared the performance of SV-MeCa with its incorporated standalone callers using two datasets, namely SVs in challenging medically relevant genes in HG002, HG002-CMRG, and SVs in autosomes of samples HG00514, HG00733, and NA19240 (Fig. 4, Supplementary Table S7). For deletions, Manta, LUMPY, and TARDIS achieved consistently higher precision values than SV-MeCa, as well as Delly for HGSVC2 reference samples. For insertions, SV-MeCa was outperformed by INSurVeyor und Manta, and for HG002-CMRG also by Delly. With respect to recall, SV-MeCa consistently outperformed all standalone caller approaches under consideration. Regarding $F_1$ scores, Manta achieved slightly higher values than SV-MeCa for deletions in the HGSVC2 reference samples (0.60 on average versus 0.58 on average), but was outperformed by SV-MeCa for deletions in the HG002-CMRG reference (0.55 versus 0.58). For insertions, SV-MeCa consistently achieved higher $F_1$ values than all standalone caller approaches under consideration.

### Ranking of consensus calls

**Fig. 5:**
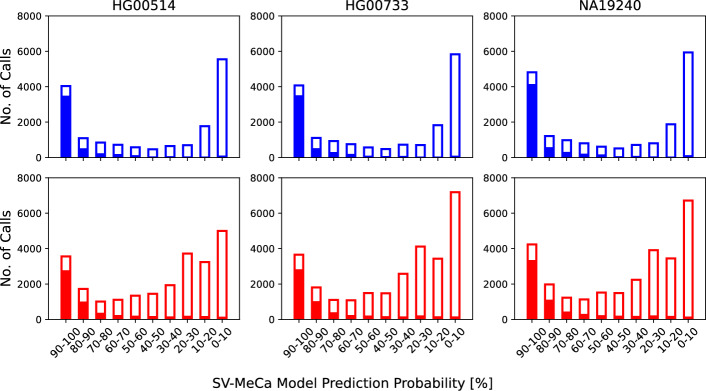
Numbers of calls per decile of SV-MeCa XGBoost prediction probability. Upper: Number of deletion calls. Lower: Number of insertion (including duplication) calls. Proportion of true positive (TP) calls is indicated by filled areas respectively

A key feature of SV-MeCa is the provision of quality values, which are retrieved from the XGBoost predicition probabilites and can be used to sort SV calls according to evidence to represent TPs. To evaluate the performance of resulting rankings, we considered SV-MeCa calls for samples HG00514, HG00733, and NA19240 per decile of assigned XGBoost prediction probabilities. Between 5550 and 5939 deletion calls per sample were assigned a probability <0.1, corresponding to 32.51$-$34.32% of all calls (Fig. 5, Supplementary Table S8). The proportion of TP calls was 0.02 for each sample. The second most frequently assigned probability decile was the highest one with between 4038 and 4817 calls, corresponding to 23.95$-$26.37% of all deletion calls. In this subgroup, the proportion of TP calls was 0.86 for each sample.

The lowest probability decile was also the most frequently assigned regarding insertions, with 5000 to 7185 calls per sample, corresponding to 20.77$-$25.74% of all calls (Fig. 5, Supplementary Table S9). The proportion of TP calls in this subgroup ranged from 0.02 to 0.03. The highest probability decile comprised between 3557 and 4236 calls (13.01$-$15.19%). In this subgroup, the proportion of TP calls ranged from 0.77 to 0.79.

### Comparison to other meta-caller approaches

**Fig. 6:**
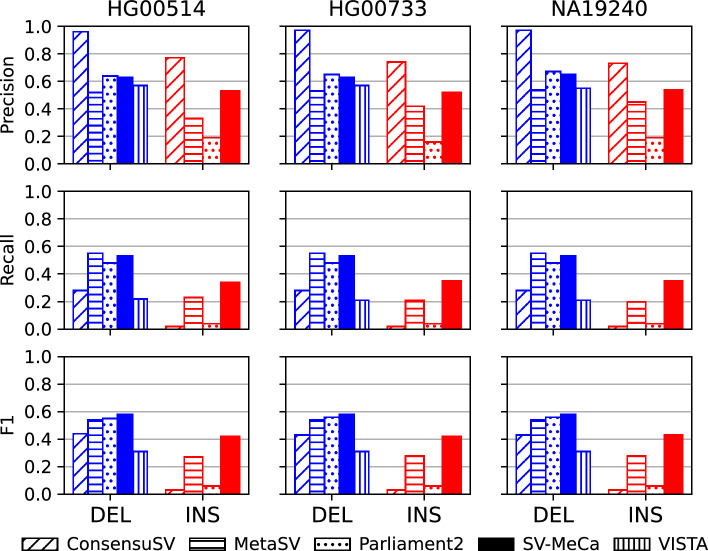
Comparison of performances of five meta-caller approaches, including SV-MeCa. Resulting values of precision, recall and $F_1$ value (Eq. 1) for reference samples HG00514, HG00733 and NA19240 using five different meta-caller approaches for structural variant calling: ConsensuSV, MetaSV, Parliament2, SV-MeCa, and VISTA. Results for deletions (DEL) are shown in blue, results for insertions including duplications (INS) are shown in red. For VISTA, only results for deletions were considered

We compared the performance of SV-MeCa with respect to recall, precision, and $F_1$ against ConsensuSV [10], MetaSV [7], Parliament2 [9], and VISTA [12] (deletions only) on samples HG00514, HG00733, and NA19240 (Fig. 6).

ConsensuSV consistently achieved the highest precision, with 0.96$-$0.97 for deletions and 0.73$-$0.77 for insertions (Supplementary Table S10). On the other hand, MetaSV achieved the highest recall for deletions (0.55 for each sample), whereas for insertions, SV-MeCa outperformed the other meta-callers with recall 0.35 on average. Finally, based on the $F_1$ score, SV-MeCa was superior to all the other meta-callers for both deletions (0.58 for each sample) and insertions (0.42 on average).

## Discussion

We presented SV-MeCa, which is to our knowledge the first meta-caller approach for SVs that incorporates individual, VCF-retrieved quality metrics into a comprehensive model for generation of the final list of consensus calls. We demonstrated the benefit of the introduced approach by comparing the results of SV-MeCa to the results of a basic XGBoost model, considering caller agreement and SV length exclusively. Further, SV-MeCa outperformed existing SV meta-caller approaches consistently with respect to $F_1$.

Based on the prediction probabilities assigned by deletion- and insertion-specific XGBoost classifiers, SV-MeCa provides a score for each SV call that indicates the evidence of representing a TP, real-world finding. The models may also allow conclusions to be drawn about which metrics must be prioritized from VCF output of established SV callers in order to distinguish TP and artifact calls as reliably as possible. For example, information about microhomologies was consistently included for both SV types (Tables 2 and 3), again emphasizing the role of microhomologies as prominent mediators of SV generation [28]. Further, Delly’s QUAL (at least indirectly as binary encoding of the PASS flag) and TARDIS’ CNVL values were consistently considered for both deletion and insertion calls, indicating the general ability of these metrics to appropriately aggregate the overall evidence for a TP SV call. A comprehensive investigation of VCF-retrieved quality metrics and their contribution to the assement of SV calls could be the subject of a future study.

It is undisputed that SV calling on short-read data is principally limited. A comprehensive evaluation by the HGSVC2 consortium revealed that approximately 30% of SVs detectable in long-read sequencing data could not be identified in Illumina short-read data [24]. Due to the known limitations, it is often argued that meaningful SV calling can and should only be applied to so-called 3rd generation, i.e., long-read sequencing data. However, in large-scale sequencing projects, and particularly in routine clinical diagnostics, short-read sequencing still represents the standard for reasons of cost, efficiency, and expertise. Nevertheless, it should be noted that consistently more than 40% of the known SVs in our benchmark data were not identified by any of the integrated standalone SV callers, and thus, contribute the vast majority of observed #FNs, rather than those misclassified by the SV-MeCa model. However, the introduced approach allows for the easy inclusion of additional (future) SV callers for recall improvement, while controlling the rate of false positives.

The development of SV callers is principally hampered by limited availability of appropriate benchmark data. All standalone SV callers included in SV-MeCa, as well as the meta-callers against which we compared its performance, were also developed and evaluated, at least in part, on the basis of data from 1000 Genomes samples. Therefore, overfitting effects can not be ruled out, besides a bias towards recurrent SVs in high confidence regions. With the rise of WGS in routine diagnostics, we will be able to broaden the data basis for SV-MeCa model training as well as its evaluation by real-world data from clinical practice. The robustness of future SV-MeCa models will benefit in particular from more variable mean coverages and read lengths in training data. The current training data from GIAB HG002 is characterized by its high quality and quite long reads (2x250), which might be uncommon in practice. The following further improvements of the SV-MeCa approach are aimed for by the developers: In addition to its extension to further SV types, such as inversions and genomic rearrangements, the objective is to assess whether the development and application of specific models for insertions and duplications will further improve SV-MeCa’s performance. Finally, we are aware that the accuracy of SV meta-callers crucially relies on the accuracy of the tools employed for SV comparison and merging. Limitations of existing tools have been reported consistently [29]. Therefore, we plan to evaluate if and how SV-MeCa’s performance may be further improved by employing alternative approaches to SV merging.

## Conclusion

SV-MeCa, which is publicly available at https://github.com/ccfboc-bioinformatics/SV-MeCa as a ready-to-use Docker container, outperforms existing SV meta-caller approaches by taking variant-specific quality metrics into account. These metrics are retrieved from individual VCF outputs of seven incorporated SV callers. The XGBoost prediction probabilities serving as scores in SV-MeCa’s VCF output, provide a suitable filter criterion to adjust the final consensus of SV calls straightforwardly and continuously to user needs in terms of sensitivity and precision.

## Additional file

Supplementary file 1.

## Data Availability

All data analyzed during this study are publicly available. Raw sequencing data of HG002 (NA24385) in FASTQ format was obtained from https://ftp-trace.ncbi.nlm.nih.gov/ReferenceSamples/giab/data/AshkenazimTrio/HG002_NA24385_son/NIST_Illumina_2x250bps/reads/. The GIAB SV ground truth of HG002, HG002_SVs_Tier1_v0.6.vcf.gz, was retrieved from https://ftp-trace.ncbi.nlm.nih.gov/ReferenceSamples/giab/release/AshkenazimTrio/HG002_NA24385_son/NIST_SV_v0.6/, and additionally, the substituted SVs in challenging medically relevant genes, HG002_GRCh37_CMRG_SV_v1.00.vcf.gz, from https://ftp-trace.ncbi.nlm.nih.gov/ReferenceSamples/giab/release/AshkenazimTrio/HG002_NA24385_son/CMRG_v1.00/GRCh37/StructuralVariant/. The corresponding specification of high confidence regions for SV benchmarking, HG002_SVs_Tier2_v0.6.bed, was downloaded from https://ftp-trace.ncbi.nlm.nih.gov/ReferenceSamples/giab/release/AshkenazimTrio/HG002_NA24385_son/NIST_SV_v0.6/. Raw 150bp paired-end WGS data of HG002, downsampled to 30x, was obtained from https://github.com/human-pangenomics/HG002_Data_Freeze_v1.0, and hg38-based SV benchmark in challenging medically relevant genes from https://ftp-trace.ncbi.nlm.nih.gov/ReferenceSamples/giab/release/AshkenazimTrio/HG002_NA24385_son/CMRG_v1.00/GRCh38/StructuralVariant/. Raw sequencing data from samples HG00514, HG00733, and NA19240 in CRAM format was retrieved from https://ftp.sra.ebi.ac.uk/vol1/run/ERR398/ERR3988781/, https://ftp.sra.ebi.ac.uk/vol1/run/ERR398/ERR3988823/, and https://ftp.sra.ebi.ac.uk/vol1/run/ERR398/ERR3989410/. Ground truth SV data for samples HG00514, HG00733, and NA19240, was downloaded from https://ftp.1000genomes.ebi.ac.uk/vol1/ftp/data_collections/HGSVC2/release/v2.0/integrated_callset/variants_freeze4_sv_insdel_alt.vcf.gz. Data used for XGBoost model training and evaluation is available at https://github.com/ccfboc-bioinformatics/SV-MeCa_data.

## Footnotes

1 https://github.com/human-pangenomics/HG002_Data_Freeze_v1.0.

2 www.internationalgenome.org.

3 https://pypi.org/project/xgboost/
