# Supplementary material for: SV-MeCa: an XGBoost-based meta-caller approach for structural variant calling from short-read data
Source: BMC Bioinformatics. 2025 Aug 20;26:218. doi: 10.1186/s12859-025-06246-6 (PMC12366149; doi:10.1186/s12859-025-06246-6)
Supplement: Supplementary file 1 [file 12859_2025_6246_MOESM1_ESM.zip › Supplementary_material.pdf]

SV-MeCa: An XGBoost-based meta-caller approach for structural variant calling from short read data

SUPPLEMENTARY MATERIAL

Rudel Christian Nkouamedjo Fankep, Arda Söylev, Anna-Lena Kobiela, Jochen Blom, Corinna Ernst, Susanne Motameny

SUPPLEMENTARY METHODS

1 Preparation of benchmark data

We aligned raw data (250bp Illumina paired reads in FASTQ format) for HG002 against the GRCh37 reference genome using DRAGEN v3.5.7 [10]. Starting from 52x, we employed the DownsampleSam utility of GATK v4.3 with read retaining probability of 0.67 (parameter `P`), resulting in mean coverage of 35x according to GATK v4.3 CollectWgsMetrics [5]. For substitution of structural variants (SVs) in challenging medically relevant genes (CMRG) [17], we removed all SVs in the GIAB HG002 Tier1 v0.6 SV benchmark overlapping with regions defined in the CMRG SV v1.00 BED file using bcftools, and then added the calls from the corresponding CMRG SV v1.00 VCF to the benchmark set.

We obtained 150bp paired-end WGS data from HG002, which was previously downsampled to 30x, from the download link provided at `https://github.com/human-pangenomics/HG002_Data_Freeze_v1.0`. Reads were trimmed from potential Illumina adapter sequences using cutadapt v1.18 under specification of `-m 35` and mapped against the GRCh38 reference genome using DRAGEN v4.2.9, achieving a mean coverage of 31x.

We retrieved PCR-free high coverage WGS data from HG00514, HG00733, and NA19240, in CRAM format from the 1000 Genomes International Genome Sample Resource (IGSR) data portal[1] , and converted to BAM using the samtools v1.17 `view` utility. Mean coverages of HG00514, HG00733, and NA1940 ranged between 30x and 31x according to GATK v4.3 CollectWgsMetrics, and read length was 150bp.

2 Standalone structural variant callers

2.1 BreakDancer

As a solely paired read (PR)-based approach, BreakDancer starts from classifying each input read pair in either normal or anomalous and then derives putative structural variants (SVs) from the identification of regions in which anomalous read pairs accumulate and are interconnected [1]. BreakDancer provides its output in a tab-separated text file in `.ctx` format, including calls of type deletion, insertion, inversion, intra-chromosomal translocation, inter-chromosomal translocation, and Unknown.

[1] www.internationalgenome.org

An inhouse Python script was used to convert .ctx entries of deletions and insertions from BreakDancer v1.4.5 output into VCF format for downstream processing. Duplications were not called by BreakDancer. Quality values, SV length, the total number of supporting reads `num_reads`, and counts of supporting reads at SV start and stop position (`Orientation1` and `Orientation2`; identical for insertions) were transferred from `.ctx` to VCF files. As BreakDancer does not provide the option to specify regions to ignore during SV calling, `bcftools view` was employed with parameter `-R` [4] to specify regions to include in final SURVIVOR input VCF in as corresponding BED file, which was generated previously based on the optionally specified regions to exclude using an inhouse script.

BreakDancer output was used exclusively in the deletion-specific classifier, as the tool called only three insertions on the entire GRCh37-based GIAB reference.

Quality values reported by BreakDancer were incorporated as covariate without further transformation. Further independent variables from BreakDancer output included in the deletion-specific classification model were the total number of supporting reads (`num_reads`) normalized by mean coverage and strand biases of supporting reads at SV start and stop position (Supplementary Table S1). In order to avoid division by zero, these values were retrieved from `Orientation1` and `Orientation2` by dividing the lower count by the higher one.

2.2 Delly

Delly's approach for SV calling consists of two main steps: (1) PR analysis and (2) split read (SR) screening of previously identified paired-end clusters for fine mapping [13]. According to the developers, Delly is particularly suitable for calling deletions, inversions, tandem duplications and translocations of lengths $\geq$300bp (github.com/dellytools/delly). Delly v.1.1.6 was run with default parameters. To convert Delly's default `.bcf` output into VCF, `bcftools view` was employed.

We observed a slightly improved performance of Delly when filtering for variant calls that passed all filters (`FILTER` flag equals `PASS`), e.g., for 35x HG002, Delly achieved F_1 scores of 0.67 (filtered) versus 0.66 (unfiltered) for deletions, and consistently 0.13 for insertions, respectively (data not shown). Therefore, to evaluate Delly's performance as a standalone caller, we always referred to the filtered set of variant calls, but all calls were included in the generation of SUVIVOR unions.

The following values in Delly's VCF were assumed to be independent of mean sequencing coverage, and hence, considered as potential covariates

without further transformation:

- MAPQ (Median mapping quality of paired-ends)
- SRMAPQ (Median mapping quality of split-reads)
- SRQ (Split-read consensus alignment quality)
- CE (Consensus sequence entropy)
- GQ (Genotype Quality)
- RDCN (Read-depth based copy-number estimate for autosomal sites)

The FILTER specifications PASS versus LowQual (Poor quality and insufficient number of PRs and SRs.), as well as FT (Per-sample genotype filter) values PASS versus LowQual, were incorporated as binary flags. Incorporation of a binary IMPRECISE (Imprecise structural variation) versus PRECISE (Precise structural variation) covariate was omitted, as IMPRECISE calls are indicated by the non-existence of corresponding SRMAPQ, SR, SRQ, and CE values.

The following values were assumed to be dependent on mean coverage, and hence, were incorporated as normalized by mean coverage estimate:

- PE (Paired-end support of the structural variant)
- SR (Split-read support)

Values of RV and RR refer to the number of high quality SRs supporting the SV or the reference, respectively, and are used to calculate variant fractions (VFs) of precise SV calls via VF = RV/(RV + RR)[2].

For imprecise SV calls, VFs arise analogously from DV (high quality variant PRs) and DR (high-quality reference PRs) via VF = DV/(DV + DR). Consequently, the correspondingly calculated values of VF were incorporated in the XGBoost model. Furthermore, the denominators of the corresponding VF computations (i.e., RV + RR and DV + DR), normalized by observed mean sequencing coverage, were included as covariate (DELLY_HQSUPPORT), as they can be considered as an estimator for local sequencing coverage. In fact, the mean of DELLY_HQSUPPORT for false positive calls ($n = 716$) was statistically significantly decreased in comparison to true positive calls ($n = 2619$, means 0.94 versus 1.19, two-sided Welch's t-test $p < 10^{-15}$, GIAB 35x benchmark sample).

[2]https://groups.google.com/g/delly-users/

The values of RC (Raw high-quality read counts or base counts for the SV), RCL (Raw high-quality read counts or base counts for the left control region), and RCR (Raw high-quality read counts or base counts for the right control region) were employed to derive a normalized read depth ratio $RD = \frac{RC}{RCL+RCR}$ to consider in model development.

The values of SVLEN (Insertion length for SVTYPE=INS) were ignored as they are already represented in the joint SURVIVOR VCF output and were retrieved from there (possibly modified).

Further, the following values in Delly's VCF output were ignored because they are expected to not provide any further evidence for the distinction between true positive, i.e., real-world and false positive SV calls:

- CIEND (PE confidence interval around END)
- CIPOS (PE confidence interval around POS)
- CHR2 (Chromosome for POS2 coordinate in case of an inter-chromosomal translocation)
- POS2 (Genomic position for CHR2 in case of an inter-chromosomal translocation)
- END (End position of the structural variant)
- CONSENSUS (Split-read consensus sequence)
- CT (Paired-end signature induced connection type)
- SVMETHOD (Type of approach used to detect SV)
- GL (Log10-scaled genotype likelihoods for RR,RA,AA genotypes)

2.3 INSurVeyor

INSurVeyor is an insertion-only caller for paired-end short-read WGS data and uses three different approaches: reference-guided assembly, de-novo assembly, and a specialised algorithm for short insertion calling [12].

INSurVeyor v.1.1.2 was executed using default settings, with the exception of adjusting the minimum size of insertions considered via the `--min-insertion-size` parameter. The output of INSurVeyor comprises two VCF files: one containing an unfiltered listing of calls (`out.vcf.gz`), and one containing only the subset of high confidence calls (`out.pass.vcf.gz`)[3].

[3]https://github.com/kensung-lab/INSurVeyor

The latter one was employed for evaluation of INSurVeyor as standalone approach. For SV-MeCa development, as well as for curation of training and evaluation data, out.vcf.gz was considered.

Filter flags ANOMALOUS_DEPTH, ALT_SHORTER_THAN_REF, LOW_SUPPORT, NO_DISC_SUPPORT, HOMOPOLYMER_INSSEQ, and NOT_ENOUGH_DISC_PAIRS in INSurVeyor's VCF output were incorporated as binary covariates in the initial model. Filter flags SMALL, ANOMALOUS_SC_NUMBER, LOW_SCORE, MH_TOO_LONG, and LOW_QUAL_ASSEMBLY were ignored, as these either did not occur at all or only once in training data.

As lengths of inserted sequences SVLEN are already extracted from SURVIVOR output, the difference between length of the inserted sequence SVINSLEN and difference in length between REF and ALT alleles SVLEN in INSurVeyor's VCF output was considered as covariate INSV_DIFF_SVLEN. Microhomology length MH_LEN was zero exclusively in training data, and therefore were ignored. Numbers of supporting discordant pairs (DISCORDANT) and split reads (SPLIT_READS), as well as spanning reads, i.e., negative evidence (SPANNING_READS), were incorporated separately per left and right breakpoint, and normalized by observed mean read depth. Strand bias of supporting split reads were retrieved using by dividing the lower count each by the higher one of the FWD_SPLIT and REV_SPLIT values per left and right breakpoint. Depths of the stable regions left and right of the insertion site predicted, were considered separately and normalized by read depth. Average edit distances of stable reads left and right in alignment to the reference sequence AVERAGE_STABLE_NM were normalized by read length (2x250bp in model training). The underlying algorithm per insertion call ALGORITHM, taking values consensus_overlap, assembly, or transurveyor was not considered, as it is implicitly represented by the assignments of OVERLAP (for consensus_overlap exclusively), SCORES and TRANS_QUERY_COV (for transuveyor exclusively), as well as SPANNING_READS and AVG_STABLE_NM (for assembly and transuveyor exclusively). The IMPRECISE flag, which is also exclusively set for calls resulting from the transuveyor approach, was incoroprated as binary flag, set to 0 if not present, irrespective of the underlying algorithm.

Flags INCOMPLETE_ASSEMBLY, FULL_JUNCTION_SEQ, and SOURCE_REGION were ignored, as they were not observed in training data.

2.4 LUMPY

The LUMPY approach incorporates evidence from read coverage, PR discordance and observed SRs, into a joint probabilistic framework per putative SV breakpoint [9]. LUMPY was run within smoove wrapper v0.2.8 (https://github.com/brentp/smoove), which is the recommended execution

mode according to its developers (https://github.com/arq5x/lumpy-sv).

Deletions (`SVTYPE=DEL`) and duplications (`SVTYPE=DUP`), treated as insertions `SVTYPE=INS`), were considered for downstream processing. According to the developers[4], novel insertions are hardly dectectable by LUMPY, and accordingly no insertion (`SVTYPE=INS`) and only 22 duplications were reported in LUMPY's VCF output for the GIAB 35x sample. Therefore, LUMPY's output was only considered in the deletion-specific classifier.

Values of `SU` (Number of pieces of evidence supporting the variant across all samples) were ignored because they correspond to the sum of `PE` (number of variant-supporting PRs) and `SR` (number of variant-supporting SRs). For LUMPY runs on a single BAM file, values of `SQ` (Phred-scaled probability that this site is variant) are identical to `QUAL`, and hence, `SQ` was not considered. Sum of quality of reference obervations `QR`, reference allele SR observation count `RS`, and reference allele PR count with partial observations recorded fractionally `RP` were incorporated as fraction of reference allele observation count with partial observations `RO`. In case $\texttt{RO} = 0$, these ratios were set to zero in order to avoid division by zero.

Concordantly, sum of quality of alternate observations `QA`, alternate allele SR observation count `AS`, alternate allele clipped-read observation count `ASC`, and alternate allele PR observation count `AP` each were incorporated as fraction of alternate allele observations `AO`.

LUMPY's VCF output provides an estimate of VF (i.e., allele balance), defined as $\texttt{AB} = \frac{\texttt{QA}}{\texttt{QR}+\texttt{QA}}$, which was considered in model development instead of genotype `GT`.

The following values in VCF output were ignored because they were expected to not provide any further evidence for the distinction between true and false positive SV calls:

- `STRANDS` (Strand orientation of the adjacency in BEDPE format); for deletions always `+-`, for duplications always `-+`
- `CIPOS`/`CIEND` (Confidence interval around `POS`/`END` for imprecise variants)
- `CIPOS95`/`CIEND95` (Confidence interval (95%) around `POS`/`END` for imprecise variants
- `GL` (Genotype Likelihood)

[4] https://github.com/arq5x/lumpy-sv/issues/160

2.5 Manta

Manta uses PR and SR information to build a graph with edges connecting genomic regions which have a possible breakend association [2]. Subsequently, graph edges or groups of highly connected edges are analyzed individually to specify putative SVs and score them, for which assigned reads are assembled and aligned back to the genome. Manta v1.6 was run with default parameters, except for specifications regarding parallelization via parameters `-j` and `-g` in `runWorkflow.py` execution and (optional) specification of the genomic regions to consider via parameter `--callRegions`. The latter requires a corresponding compressed BED file as additional input, which is generated automatically prior to program call based on the optionally specified regions to exclude using an inhouse script.

We consistenly considered the entire set of filtered (`FILTER` equals `PASS`) and unfiltered (`FILTER` unequals `PASS`) calls in output file `diploidSV.vcf.gz`.

The fractions of observed alternate allele supporting SRs and the sum of alternate and reference supporting SRs (encoded by the `SR` tag) and the fractions of observed alternate allele supporting PRs and the sum of alternate and reference supporting PRs (encoded by the `PR` tag), were replaced by a joint estimate of VF based on SRs and PRs, i.e., the sum of alternate allele-supporting SRs and PRs divided by the overall sum of SRs and PRs (MANTA_VF). Furthermore, counts of observed alternate allele-supporting SRs and PRs were incorporated, each normalized by observed mean coverage (MANTA_SR, MANTA_PR).

Manta's VCF output provides the following fields in the `FILTER` column:

- `MinQUAL` (`QUAL` score is less than 20)
- `MinSomaticScore` (`SOMATICSCORE` is less than 30, does only apply for somatic SV calling)
- `Ploidy` (only for DEL and DUP variants: genotypes of overlapping variants (with similar size) are inconsistent with diploid expectation)
- `MaxDepth` (depth is greater than 3x the median chromosome depth near one or both variant breakends)
- `MaxMQ0Frac` (only for small SVs <1000bp: fraction of reads in all samples with `MAPQ0` around either breakend exceeds 0.4)
- `NoPairSupport` (for variants significantly larger than the PR fragment size: no PRs support the alternate allele)
- `SampleFT` (sample-specific filters `MinGQ` or `HomRef` not passed)

The FILTER value is PASS if all of the aforementioned filters were passed. As covariates for the XGboost classifier, binary flags for passing the MaxMQ0Frac, NoPairSupport, and SampleFT filter were employed in the deletion-specific model. For insertion calls originating from 35x GIAB data, MaxMQ0Frac was constant zero and NoPairSupport only once not zero. Hence, these values from Manta's VCF output were not incorporated in development of the insertion-specific classifier. SV calls not passing the Ploidy filter occured only twice and only in deletion-specific input data for model training based on 35x GIAB, therefore this filter had to be ignored in model development. The remaining FILTER tags were ignored as they do either not apply for untargeted germline SV calling, or are already reflected in the QUAL and MAPQ values. Concordantly, the FT value in Manta's VCF output was ignored, as it is almost equivalent to SampleFT.

2.6 Pindel

The Pindel approach uses PRs of which only one read could be mapped to the reference genome as starting point, and then applies a pattern growth data structure for exact string matching to determine the putative mapping locations of the splitted terminals of the unmapped read [19]. Pindel is able to detect deletions, insertions, inversions and tandem duplications, but the range in which the size of an SV must lie in order to be detectable in principle, depends crucially on read length and fragment (i.e., RP insertion) size [18]. However, the length of detectable deletions is theoretically unlimited, unless specified otherwise by the user.

Pindel v0.2.5b9 was primarily executed following the recommendations of its developers. This entailed specifying the expected RP insert size and generating VCF files using the bundled utilities `pindel` and `pindel2vcf`. An estimation of the insert size was obtained from the BreakDancer's `*.ctx` output file. Since Pindel's sensitivity is high, minor adjustments were made. These adjustments included modifying the minimum number of reads required to support a call via the parameter `-M`, activating the germline mode through the parameter `-N`, and optimizing runtime performance by deactivating the reporting of inversions via the parameter `-r`, as inversions are not within the current tool's scope.

Additionally, Pindel was configured to run chromosome-wise using the parameter `-c`, and regions to exclude were specified via the parameter `-J`.

Pindel VCF output does not provide individual quality scores per call, but number of reads supporting the alternative and the reference allele, respectively. Therefore, the ratio of local coverage, i.e., the sum of comma-separated AD values, and observed mean coverage, as well as the ratio of

alternate allele supportive reads and the sum of comma-separated `AD` values as an estimator of VF, were considered as potential covariates (PINDEL_VF) in XGBoost model development.

2.7 TARDIS

TARDIS provides a largely automated framework for detection and characterization of deletions, inversions, novel insertions and duplications (including probabilistic models specifcally adapted for direct, inverted, and tandem duplications each), based on whole-genome short read data [14, 15]. In a nutshell, the underlying algorithm identifies putative SV clusters based on discordantly mapped PRs and SRs, and then aims to minimize their total number based on maximum parsimony. Finally, SR and RD signatures are used to assign a likelihood score to each SV call in order to identify putative false positives. TARDIS includes genome-specific information on known segmental duplications, repeats, gaps, satellite regions, mobile elements and GC contents via pre-built Some Organism's Nucleotide Information Container (SONIC) files in the calling process. Additional regions to ignore can be passed in bed format via the `--gap` parameter[5]. TARDIS produces a VCF file including information on number of supporting PRs and SRs, length and type per SV call, among others. According to the developer, however, the given `QUAL` value is (so far) hard-coded. We consistently condiered the enti

We considered the entire set of filtered (`FILTER` equals `PASS`) and unfiltered (`FILTER` equals `mfilt`) SV calls in TARDIS' VCF output for all analyses, as we consistently observed a decrease of resulting F_1 scores when only filtered calls were included instead of all calls (data not shown).

SV-MeCa runs TARDIS v1.0.8 as recommended by the developers, i.e., after elimination of PCR duplicates in the input bam file with Sambamba v1.0 (https://lomereiter.github.io/sambamba/) and optional specification of regions to exclude via `--gap`. The original VCF output was sorted using awk v4.0.2 to be processable for SURVIVOR.

Besides quality score `CNVL`, the predicted genotype `GT` (encoded via `0/0` $\rightarrow$ 0; `0/1` $\rightarrow$ 0.5; `1/1` $\rightarrow$ 1), the ratio of supporting paired reads (`RPSUP`), respectively split reads (`SRSUP`), and observed mean coverage were considered as covariates in XGboost model development.

The binary flag of the `PRECISE` (1) versus `IMPRECISE` (0) flag took value 1 exclusively for deletion calls and value 0 exclusively for insertion calls, and therefore was not considered in model development

[5]https://github.com/BilkentCompGen/tardis/issues/14

3 Processing standalone caller VCF outputs & running SURVIVOR

We first filtered individual SV calls for insertions (including duplications) and deletions with length ≥50bp using our inhouse scripts. Then we replaced `SVTYPE=DUP` with `SVTYPE=INS` in the VCF output of Delly, LUMPY, Manta, Pindel, and TARDIS (BreakDancer does not report duplications) for SURVIVOR input and discarded Pindel's SV calls labeled with `SVTYPE=RPL`. The `merge` utility of SURVIVOR v1.0.7 [8] was run under specification of a minimum required overlap of 90% of individual SV lengths for the generation of training data. To account for SURVIVOR's over-merging habit, which has been described by the developers of the Truvari [6] and in accordance with the parameters used by SV-MeCa, SURVIVOR was called under specification of 50bp as the maximum allowed distances between corresponding start and stop positions of overlapping SV calls for evaluation. Note that number of supporting callers and the strand information were discarded as a matter of principle.

4 Feature extraction & transformation

4.1 General approach

For each (consensus) SV call in the corresponding SURVIVOR output, we only extracted the absolute mean number of affected nucleotides, i.e., absolute SV length encoded by the `SVLEN` tag. All the remaining quality metrics considered in XGBoost model development were extracted directly from the VCF output of standalone callers. For merged SV calls in SURVIVOR's VCF output originating from the same tool, we parsed the call with the most favourable overall quality estimate for the model input, i.e., with maximum `Score` value in BreakDancer's `.ctx` output, with maximum `QUAL` value in Delly's, LUMPY's, and Manta's VCF output, or minimum `CNVL` value in TARDIS' VCF output. For Pindel, which does not provide a comparable overall quality estimate, we extracted covariates from the call with the highest count of alternate allele-supporting reads, as encoded by the `AD` values.

As a matter of principle, we discarded the confidence intervals around the start and stop positions of SVs (encoded by `CIPOS` in the output VCF of Delly, LUMPY, Manta and TARDIS) and genotype likelihoods. Furthermore, we generally replaced genotypes `0/0`, `0/1`, and `1/1` with estimators of variant fraction, which was not possible for BreakDancer and TARDIS.

For the development of the deletion-specific model, we initially considered

51 potential covariates; including 4 for BreakDancer, 15 for Delly, 14 for LUMPY, 10 for Manta, 3 for Pindel, and 4 for TARDIS. Whereas for the insertion calls, we considered 56 covariates; including 15 for Delly, 25 for INSurVeyor, 8 for Manta, 3 for Pindel, and 4 for TARDIS.

4.2 Tranformation of quality scores

BreakDancer, Delly, LUMPY, and Manta provide individual quality scores per call, encoded as `QUAL` in VCF output and as `Score` in BreakDancer's `*.ctx` file output. To assess the dependence of these quality scores on mean coverage, simple linear regression analyses using ordinary least squares with corresponding values of concordant calls resulting from the 52x and the 35x GIAB sample, were applied, both for original values, as well as for their normalized rank scores (Supplementary Fig. S1, Supplementary Fig. S2). As the quality scores were constrained by maximum values for three tools (BreakDancer `Score` ≤ 99; Delly `QUAL` $\leq 10^4$; Manta `QUAL` ≤ 999), corresponding observations were excluded from regression analyses, regardless of whether concerning the dependent or the independent variable, to avoid bias.

Regarding BreakDancer, quality values were found to be independent of read coverage, i.e., linear regression yielded a slope of 0.99. Therefore, quality values reproted by BreakDancer were directly incorporated. We also did not apply any coverage-dependent transformation to TARDIS predictions, whose likelihood score `CNVL` decreases when the likelihood of a true SV call increases [16].

Quality values `QUAL` reported by Delly and LUMPY were found to be dependent on sequencing depth, and hence, normalized rank scores of `QUAL` were incorporated into model development. Quality values `QUAL` provided by Manta for deletions were found to be linearly dependent on sequencing depth (Supplementary Figure S1), and hence, were normalized by observed mean coverage. For insertions, the linear relationship was not clearly evident (Supplementary Figure S2), but for consistency reasons, the same relationship as for deletions was assumed. Considering the 35x GIAB reference data set, the maximum quality value (i.e., `QUAL`=999) was assigned to a noticeable amount of 38.36% (1259/3282) of Manta's SV calls. Given an input sample with sequencing depth >35, due to the coverage-dependent transformation, all of Manta's SV calls would be assigned to `QUAL` values <999. In order to keep the original proportion of SV calls with maximum `QUAL`, corresponding values were excluded from transformation. On the other hand, given input data with sequencing depth < 35, the transformation yields `QUAL` values greater than maximum value 999. These values were reset to the maximum value 999.

INSurVeyor does not report a consistent quality value per call.

4.3 Correlation-based feature selection

In order to define the set of covariates to be considered for the SV-MeCa XGBoost classifier training, we applied a correlation-based feature selection: Spearman's rank correlation coefficient ρ was determined for each pair of potential features originating from the VCF output per standalone caller. For each pair with absolute $\rho > 0.6$, we only further considered the feature showing a higher correlation with the dependent variable, i.e., binary encoding of true positive observations, based on absolute ρ.

5 Hyperparameter tuning

For hyperparameter tuning, the `GridSearchCV` utility of scikit-learn v1.1.1 [11] under specification of F_1 score as scoring function was employed, i.e., a predefined hyperparameter space was exhaustively searched for the optimal combination of values with respect to maximum F_1 achieved.

The following parameters (possible assignments are shown in brackets) were included in a 5-fold cross-validated grid search, resulting in a cross-validated grid search over 798,600 combinations of values per model and a total of 3,993,00 model fits:

- learning rate $\eta \in [0 \ldots 1]$ (values from 0.05 up to and including 1.0, in steps of 0.05)
- minimum loss reduction $\gamma \in [0 \ldots \infty]$ (0, 0.05, 0.1, 0.2, 0.4, 0.8, 1.6, 3.2, 6.4, 12.8, 25.6)
- maximum depth of trees (number of covariates)
- number of trees/iterations $\in \mathbb{N}$ (values from 10 up to and including 300, in steps of 10)
- L1 regularization term on weights $\alpha \in [0 \ldots \infty]$ (0, 0.05, 0.1, 0.2, 0.4, 0.8, 1.6, 3.2, 6.4, 12.8, 25.6)
- L2 regularization term on weights $\lambda \in [0 \ldots \infty]$ (0, 0.05, 0.1, 0.2, 0.4, 0.8, 1.6, 3.2, 6.4, 12.8, 25.6)

6 Structural variant meta-callers

6.1 Parliament2

Latest version of Parliament2 Docker image (v0.1.11) [20] was obtained from DockerHub[6]. The Docker image was run with additional arguments `--breakdancer`, `--manta`, `--cnvnator`, `--lumpy`, `--delly_deletion`, `--delly_insertion`, `--delly_duplication`, and `--genotype`. Breakseq2 was omitted, as it may only be able to work when using the hs37d5 reference genome, according to the developers[7].

6.2 ConsensuSV

The latest version of the ConsensuSV v1.1 Docker image was obtained from DockerHub[8]. ConsensuSV is capable of processing both BAM and VCF files and can incorporate a total of eight SV callers. Results from five of them, namely BreakDancer, Delly, LUMPY, Manta, and TARDIS, were adopted from SV-MeCa output, whereas CNVnator results were obtained from previous Parliament2 runs. The remaining two SV callers, Breakseq and Wham, were executed specially to enable ConsensuSV to start from VCF input. Breakseq and Wham were run within the Docker container under specification of the arguments provided by the developers for execution of their pipeline starting from BAM files. Finally, ConsensuSV was executed using the model within the container, starting from VCFs with default parameters.

6.3 MetaSV

The latest version of MetaSV v0.5.4 was installed using Conda from Anaconda[9]. The script `run_metasv.py` was executed as recommended by the developers, providing VCF files from standalone SV callers using parameters such as `--pindel_vcf`, `--breakdancer_vcf`, `--breakseq_vcf`, `--cnvnator_vcf`, `--manta_vcf`, `--lumpy_vcf`, and `--cnvkit_vcf`. To enable a comparison with a comparable tool, the VCF resulting from TARDIS was used in meta-calling instead of CNVkit output. Furthermore, mean read coverage was adjusted via `--mean_read_coverage`, and final output was restricted to autosomes via the `--chromosomes` parameter.

[6] https://hub.docker.com/r/dnanexus/parliament2/

[7] https://github.com/slzarate/parliament2

[8] https://hub.docker.com/r/mateuszchilinski/consensusv-nf-pipeline

[9] https://anaconda.org/bioconda/metasv

6.4 VISTA

VISTA was obtained from Github[10] and requires VCF output from Delly, Genome STRiP, Lumpy, Manta, and Octopus as input. Input from Delly, LUMPY and Manta were adopted from SV-MeCa output. The Genome STRiP v2.1.1 software [7] was retrieved from the corresponding download page[11] and executed in a Docker container (v2.00.1833) from Biocontainers[12]. Genome STRiP was execued analogous to the calls provided in test script `svtoolkit/installtest/discovery.sh` of the software's tarball file. The most recent version of Octopus [3], v0.7.4, was executed within a Docker container retrieved from DockerHub[13] using default arguments.

VCF outputs from Delly, Lumpy, Manta and Octopus were further processed using the corresponding scripts provided within the VISTA framework[14]. In VCF output of Genome STRiP, SV lengths encoded by the `SVLEN` tag were turned into absolute values prior to running `vista.py` with default arguments.

The native output of VISTA was slightly adapted according to the requirements of Truvari `bench`, including sorting, splitting of multi-allelic sites with BCFTool's `norm` utility, and renaming chromosomes by adding prefix `chr`. The script for executing VISTA, processing VCF outputs, and running Truvari's `bench` utility is provided in the `SV-MeCa_data` GitHub repository[15].

7 Runtime

Starting from BAM files, SV-MeCa's runtime ranged from 10.8 to 13.3 hours per processed HGSVC2 sample on a notebook with 20 threads and 64GB RAM.

References

[1] K. Chen, J. W. Wallis, M. D. McLellan, D. E. Larson, J. M. Kalicki, C. S. Pohl, S. D. McGrath, M. C. Wendl, Q. Zhang, D. P. Locke, et al. Breakdancer: an algorithm for high-resolution mapping of genomic structural variation. *Nature methods*, 6(9):677–681, 2009.

[10] https://github.com/Mangul-Lab-USC/VISTA

[11] https://software.broadinstitute.org/software/genomestrip/download-genome-strip

[12] https://biocontainers.pro/tools/genomestrip

[13] https://hub.docker.com/r/dancooke/octopus

[14] https://github.com/Mangul-Lab-USC/VISTA/tree/main/scripts

[15] https://github.com/ccfboc-bioinformatics/SV-MeCa_data/vista

[2] X. Chen, O. Schulz-Trieglaff, R. Shaw, B. Barnes, F. Schlesinger, M. Källberg, A. J. Cox, S. Kruglyak, and C. T. Saunders. Manta: rapid detection of structural variants and indels for germline and cancer sequencing applications. *Bioinformatics*, 32(8):1220–1222, 2016.

[3] D. P. Cooke, D. C. Wedge, and G. Lunter. A unified haplotype-based method for accurate and comprehensive variant calling. *Nature Biotechnology*, 2021.

[4] P. Danecek, J. K. Bonfield, J. Liddle, J. Marshall, V. Ohan, M. O. Pollard, A. Whitwham, T. Keane, S. A. McCarthy, R. M. Davies, et al. Twelve years of samtools and bcftools. *Gigascience*, 10(2):giab008, 2021.

[5] M. A. DePristo, E. Banks, R. Poplin, K. V. Garimella, J. R. Maguire, C. Hartl, A. A. Philippakis, G. Del Angel, M. A. Rivas, M. Hanna, et al. A framework for variation discovery and genotyping using next-generation dna sequencing data. *Nature genetics*, 43(5):491–498, 2011.

[6] A. C. English, V. K. Menon, R. A. Gibbs, G. A. Metcalf, and F. J. Sedlazeck. Truvari: refined structural variant comparison preserves allelic diversity. *Genome Biology*, 23(1):271, 2022.

[7] R. E. Handsaker, V. Van Doren, J. R. Berman, G. Genovese, S. Kashin, L. M. Boettger, and S. A. McCarroll. Large multiallelic copy number variations in humans. *Nature genetics*, 47(3):296–303, 2015.

[8] D. C. Jeffares, C. Jolly, M. Hoti, D. Speed, L. Shaw, C. Rallis, F. Balloux, C. Dessimoz, J. Bähler, and F. J. Sedlazeck. Transient structural variations have strong effects on quantitative traits and reproductive isolation in fission yeast. *Nature communications*, 8(1):14061, 2017.

[9] R. M. Layer, C. Chiang, A. R. Quinlan, and I. M. Hall. Lumpy: a probabilistic framework for structural variant discovery. *Genome biology*, 15(6):1–19, 2014.

[10] N. A. Miller, E. G. Farrow, M. Gibson, L. K. Willig, G. Twist, B. Yoo, T. Marrs, S. Corder, L. Krivohlavek, A. Walter, et al. A 26-hour system of highly sensitive whole genome sequencing for emergency management of genetic diseases. *Genome medicine*, 7:1–16, 2015.

[11] F. Pedregosa, G. Varoquaux, A. Gramfort, V. Michel, B. Thirion, O. Grisel, M. Blondel, P. Prettenhofer, R. Weiss, V. Dubourg, et al. Scikit-learn: Machine learning in python. *the Journal of machine Learning research*, 12:2825–2830, 2011.

[12] R. Rajaby, D.-X. Liu, C. H. Au, Y.-T. Cheung, A. Y. T. Lau, Q.-Y. Yang, and W.-K. Sung. Insurveyor: improving insertion calling from short read sequencing data. *Nature Communications*, 14(1):3243, 2023.

[13] T. Rausch, T. Zichner, A. Schlattl, A. M. Stütz, V. Benes, and J. O. Korbel. Delly: structural variant discovery by integrated paired-end and split-read analysis. *Bioinformatics*, 28(18):i333–i339, 2012.

[14] A. Soylev, C. Kockan, F. Hormozdiari, and C. Alkan. Toolkit for automated and rapid discovery of structural variants. *Methods*, 129:3–7, 2017.

[15] A. Soylev, T. M. Le, H. Amini, C. Alkan, and F. Hormozdiari. Discovery of tandem and interspersed segmental duplications using high-throughput sequencing. *Bioinformatics*, 35(20):3923–3930, 2019.

[16] A. Soylev, T. M. Le, H. Amini, C. Alkan, and F. Hormozdiari. Discovery of tandem and interspersed segmental duplications using high-throughput sequencing. *Bioinformatics*, 35(20):3923–3930, 2019.

[17] J. Wagner, N. D. Olson, L. Harris, J. McDaniel, H. Cheng, A. Fungtammasan, Y.-C. Hwang, R. Gupta, A. M. Wenger, W. J. Rowell, et al. Curated variation benchmarks for challenging medically relevant autosomal genes. *Nature biotechnology*, 40(5):672–680, 2022.

[18] K. Ye, L. Guo, X. Yang, E.-W. Lamijer, K. Raine, and Z. Ning. Split-read indel and structural variant calling using pindel. *Copy Number Variants: Methods and Protocols*, pages 95–105, 2018.

[19] K. Ye, M. H. Schulz, Q. Long, R. Apweiler, and Z. Ning. Pindel: a pattern growth approach to detect break points of large deletions and medium sized insertions from paired-end short reads. *Bioinformatics*, 25(21):2865–2871, 2009.

[20] S. Zarate, A. Carroll, M. Mahmoud, O. Krasheninina, G. Jun, W. J. Salerno, M. C. Schatz, E. Boerwinkle, R. A. Gibbs, and F. J. Sedlazeck. Parliament2: Accurate structural variant calling at scale. *GigaScience*, 9(12):giaa145, 2020.

SUPPLEMENTARY TABLES

Supplementary Table S1: Covariates considered in XGBoost classifier training. For development of the deletion-specific model, 51 covariates were initially considered, 16 of which were discarded due to pairwise Spearman's rank correlation coefficient $\rho > 0.6$. 31 coavariates were considered in the final SV-MeCa model. For development of the XGBoost classifier for insertions including duplications, 56 covariates were initially considered, 20 of which were discarded due to $\rho > 0.6$. 34 coavariates were considered in the final SV-MeCa model. Corr: Correlation; DEL: Deletion; INS: Insertion.

Supplementary Table S2: Counts of true positive (#TP), false positive (#FP), and false negative (#FN) structural variant (SV) calls, and resulting precision, recall and F_1 values, given the Genome in a Bottle (GIAB) reference data in 52x HG002 per standalone caller, and for the SURVIVOR-derived union of all calls, subdivided by deletions (DEL) and insertions including duplications (INS). For the evaluation of Delly and INSurVeyor as standalone approaches, only high confidence calls were considered (see Supplementary Methods).

Supplementary Table S3: Optimal assignment of hyperparameters in deletion- and insertion-specific XGBoost classifiers, due to hyperparameter tuning via grid search.

Supplementary Table S4: Counts of true positive (#TP), false positive (#FP), true negative (#TN), and false negative (#FN) classifications, as well as resulting accuracies, of the initial and the final SV-MeCa full models on training data. #covariates refers to the number of covariates considered per model, and N to the total number of classified observations. DEL: Deletions; INS: Insertions.

Supplementary Table S5: Counts of true positive (#TP), false positive (#FP), true negative (#TN), and false negative (#FN) classifications, as well as resulting accuracies, on the SURVIVOR-derived union of structural variant calls in HGSVC2 reference samples HG00514, HG00733, and NA19240, stratified by deletions (DEL) and insertions including duplications (INS). BM: Basic model; FM: Full model.

Supplementary Table S6: Counts of true positive (#TP), false positive (#FP), and false negative (#FN) classifications, and resulting values of precision, recall and F_1, for HGSVC2 reference samples HG00514, HG00733, and NA19240, stratified by deletions (DEL) and insertions including duplications (INS). BM: Basic model; FM: Full model.

Supplementary Table S7: Counts of true positive (#TP), false positive (#FP), and false negative (#FN) calls achieved by standalone callers BreakDancer (deletions only), Delly, INSurVeyor (insertions only), LUMPY (deletions only), Manta, Pindel, and TARDIS, as well as SV-MeCa, and resulting values of precision, recall and F_1, for structural variants in challenging medically relevant genes in HG002 (HG002-CMRG) and HGSVC2 reference samples HG00514, HG00733, and NA19240, stratified by deletions (DEL) and insertions including duplications (INS).

Supplementary Table S8: Numbers of assigned calls (N), as well as number of true positve calls (#TP) contained therein, per decile of prediction probability assigned by SV-MeCa's XGBoost classifier for deletions.

Supplementary Table S9: Numbers of assigned calls (N), as well as number of true positve calls (#TP) contained therein, per decile of prediction probability assigned by SV-MeCa's XGBoost classifier for insertions including duplications.

Supplementary Table S10: Performance of ConsensuSV, MetaSV, Parliament2, SV-MeCa, and VISTA, with respect to precision, recall, and F_1 for HGSVC2 reference samples HG00514, HG00733, and NA19240, stratified by deletions (DEL) and insertions including duplications (INS). For VISTA, only deletions were considered.

SUPPLEMENTARY FIGURES

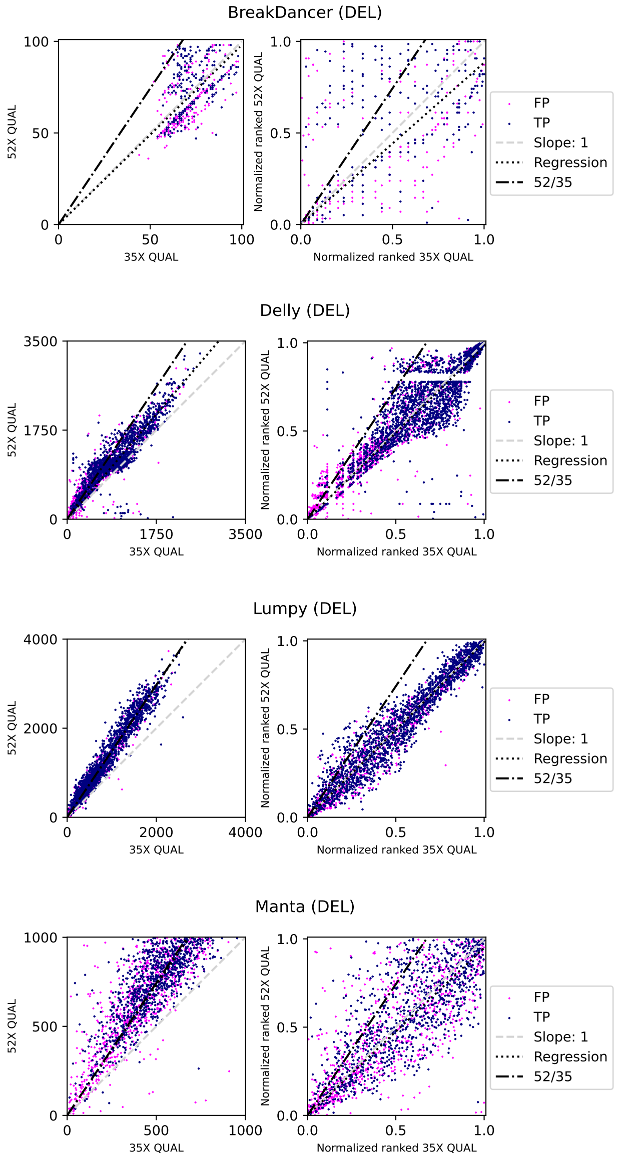

Supplementary Figure S1: Quality values for concordant deletion calls for the HG002/NA24385 35x and 52x benchmark samples of standalone structural variant (SV) callers BreakDancer, Delly, LUMPY, and Manta. Original values are shown on the left, normalized rank scores on the right plots. Maximum quality values of 99 for BreakDancer, 10^4 for Delly, and 999 for Manta have been removed. FP: false positive SV calls; TP: true positive SV calls.

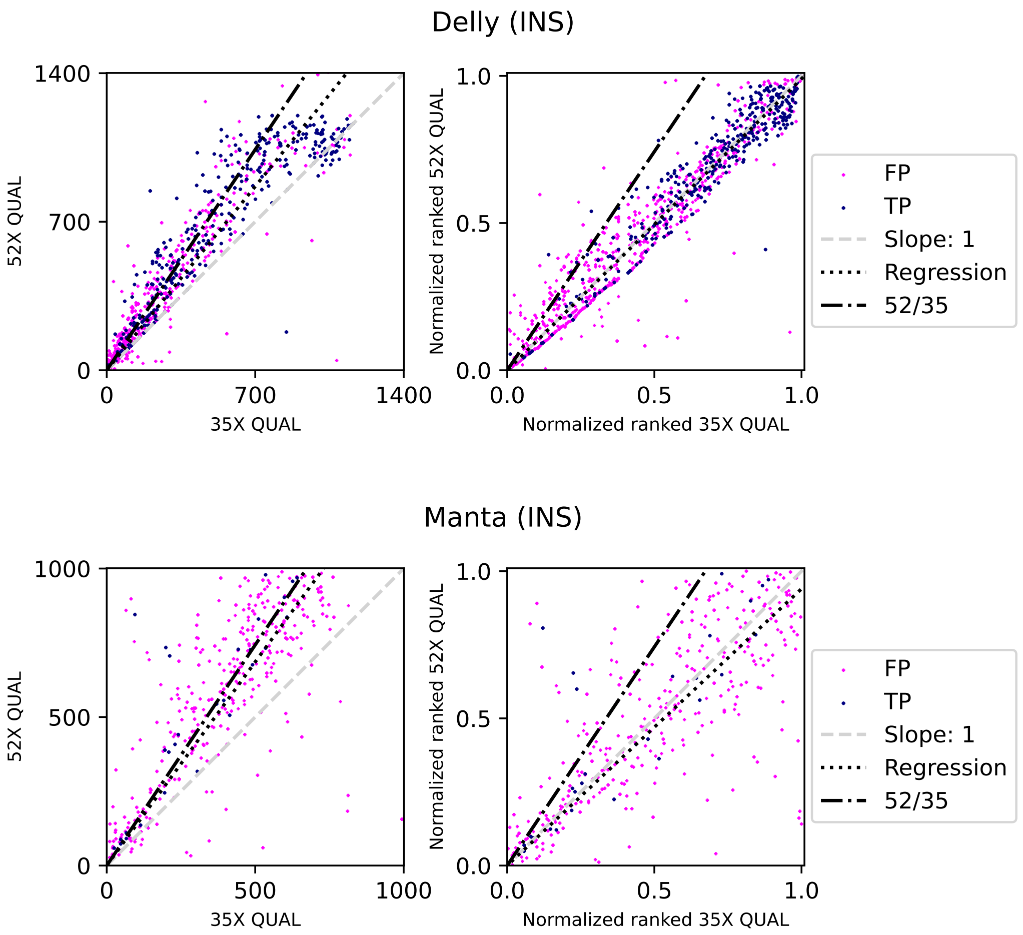

Supplementary Figure S2: Quality values for concordant insertion (including duplication) calls for the HG002/NA24385 35x and 52x benchmark samples of standalone structural variant (SV) callers Delly and Manta. Original values are shown on the left, normalized rank scores in the right plots. Maximum quality values of 99 for BreakDancer, 10^4 for Delly, and 999 for Manta had been removed. FP: false positive SV calls; TP: true positive SV calls.

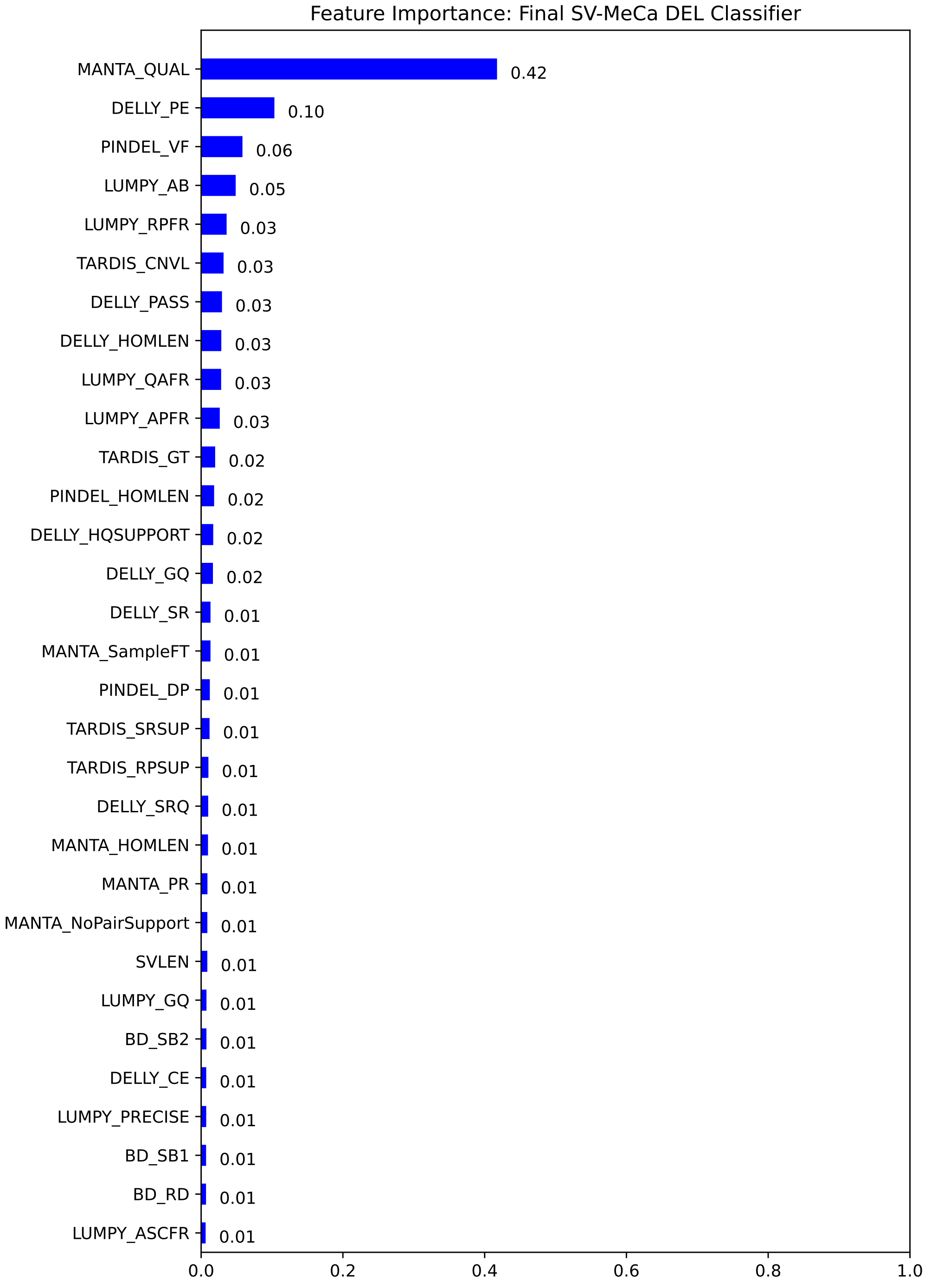

Supplementary Figure S3: Feature importance, i.e., gain (the relative contribution of the corresponding feature to the model), of 31 covariates included in the deletion-specific SV-MeCa XGboost classifier.

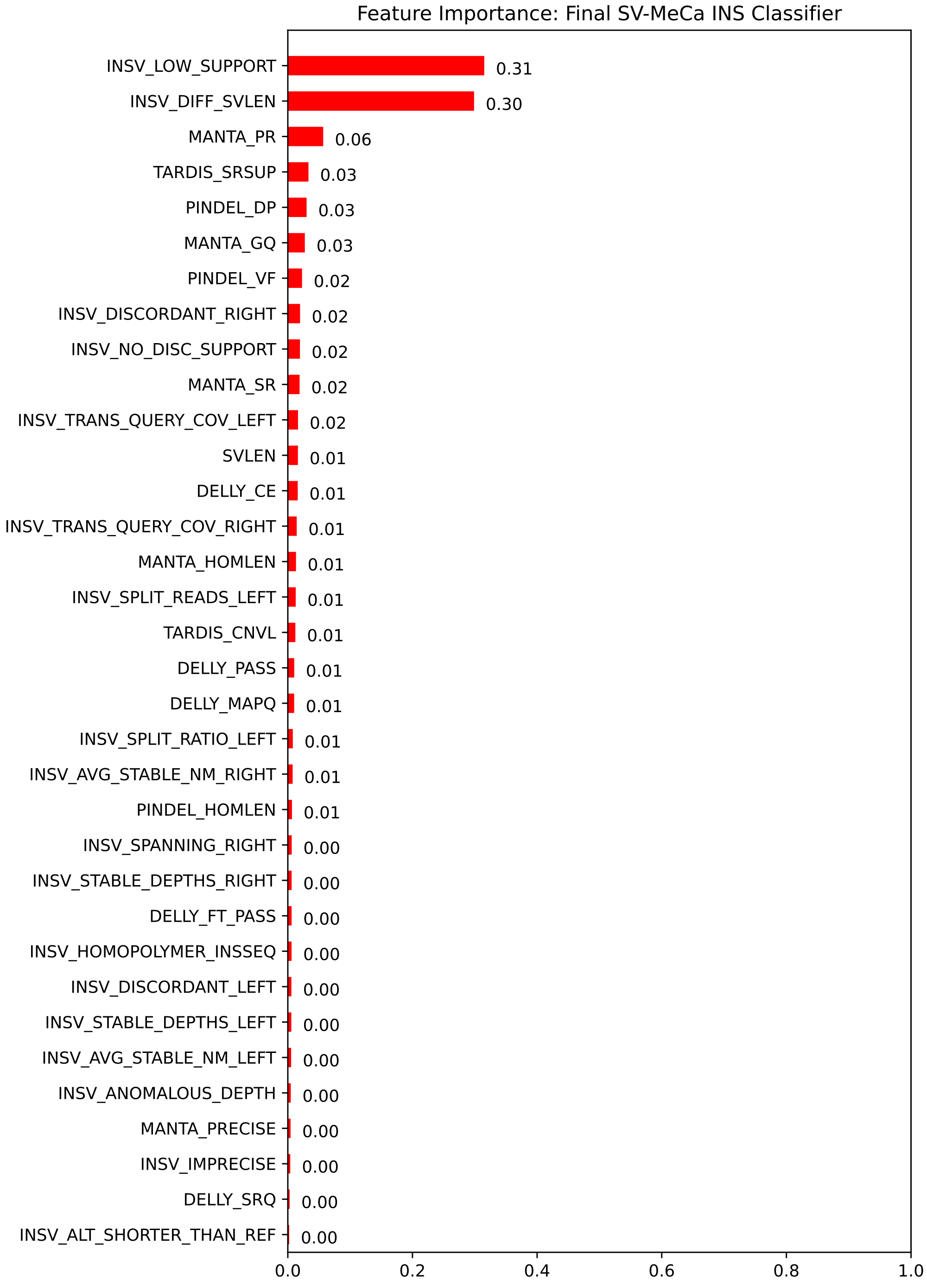

Supplementary Figure S4: Feature importance, i.e., gain (the relative contribution of the corresponding feature to the model), of 34 covariates included in the insertion-specific SV-MeCa XGboost classifier.

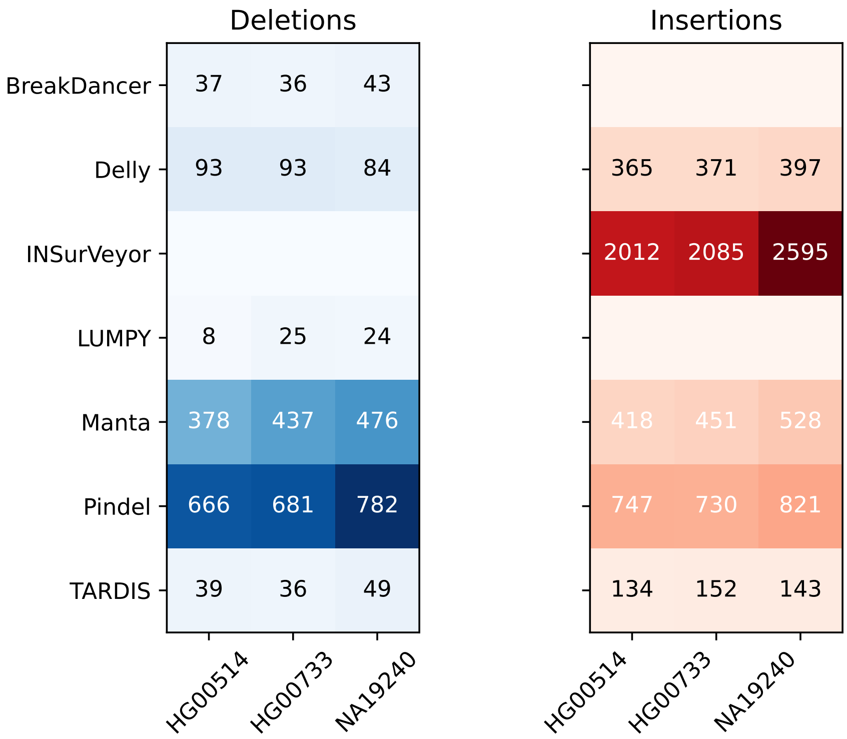

Supplementary Figure S5: Number of exclusive, true positive structural variant calls per tool and HGVSC2 reference sample in SURVIVOR union, stratified by deletions and insertions (including duplications).
